# Risk Factors for Medication-Related Osteonecrosis of the Jaw—A Binomial Analysis of Data of Cancer Patients from Craiova and Constanta Treated with Zoledronic Acid

**DOI:** 10.3390/jcm12113747

**Published:** 2023-05-29

**Authors:** George Adrian Ciobanu, Adrian Camen, Mihaela Ionescu, Daniel Vlad, Cristina Maria Munteanu, Mircea Ionuț Gheorghiță, Cristian Virgil Lungulescu, Ionela Elisabeta Staicu, Elena Claudia Sin, Luminița Chivu, Răzvan Mercuț, Sanda Mihaela Popescu

**Affiliations:** 1 Department of Oral Rehabilitation, University of Medicine and Pharmacy of Craiova, 200349 Craiova, Romania; 2 Department of Oral and Maxillofacial Surgery, Dental Medicine Faculty, “Ovidius” University of Constanța, 900470 Constanța, Romania; 3 Department of Oral and Maxillofacial Surgery, University of Medicine and Pharmacy of Craiova, 200349 Craiova, Romania; 4 Department of Medical Informatics and Biostatistics, University of Medicine and Pharmacy of Craiova, 200349 Craiova, Romania; 5 Department of Oncology, University of Medicine and Pharmacy of Craiova, 200349 Craiova, Romania; 6 Department of Orthodontics, University of Medicine and Pharmacy of Craiova, 200349 Craiova, Romania; 7 Department of Oral and Maxillofacial Surgery, The County Emergency Clinical Hospital “Sf. Apostol Andrei”, 900591 Constanța, Romania; 8 Department of Plastic Surgery, University of Medicine and Pharmacy of Craiova, 200349 Craiova, Romania

**Keywords:** osteonecrosis, bisphosphonate, zoledronic acid, risk factors

## Abstract

MRONJ (Medication-Related Osteonecrosis of the Jaw) is a condition observed in a subset of cancer patients who have undergone treatment with zoledronic acid in order to either prevent or treat bone metastases. The primary aim of this research was to establish the importance of risk factors in the development of medication-related osteonecrosis of the jaw in cancer patients receiving zoledronic acid therapy for bone metastases. The present study is an observational retrospective investigation conducted at two university centers, namely, Craiova and Constanța, and included cancer patients treated with zoledronic acid. The medical records of the patients were obtained over a four-year timeframe spanning from June 2018 to June 2022. The data analysis was carried out between January 2021 and October 2022. Patients were treated for cancer, bone metastases, and MRONJ according to the international guidelines. The research investigated a cohort of 174 cancer patients (109 females and 65 males) aged between 22 and 84 years (with a mean age 64.65 ± 10.72 years) seeking treatment at oncology clinics situated in Craiova and Constanța. The study conducted a binomial logistic regression to analyze ten predictor variables, namely, gender, age, smoking status, treatment duration, chemotherapy, radiotherapy, endocrine therapy, presence of diabetes mellitus (DM), obesity, and hypertension (HT). The results of the analysis revealed that only five of the ten predictor variables were statistically significant for MRONJ occurrence: duration of treatment (*p* < 0.005), chemotherapy (*p* = 0.007), and hypertension (*p* = 0.002) as risk factors, and endocrine therapy (*p* = 0.001) and obesity (*p* = 0.024) as protective factors.

## 1. Introduction

The condition known as BRONJ (Bisphosphonate-Related Osteonecrosis of the Jaw) [1,2], MRONJ (Medication-Related Osteonecrosis of the Jaw) [3,4], or DIONJ (Drug-induced Osteonecrosis of the Jaw) [5] is characterized by debilitating symptoms, with profound effects on patients’ quality of life [6,7]. MRONJ occurs in cancer patients at risk of bone metastases, who have undergone treatment with antiresorptive medications such as bisphosphonates or denosumab, as well as anti-angiogenic agents, monoclonal antibodies, or other drugs [3,4]. MRONJ is defined by the AAOMS (American Association of Oral and Maxillofacial Surgeons) as a necrotic bone exposure in the maxillofacial region that persists for at least eight weeks in a patient who has been subject to antiresorptive or antiangiogenic drugs and without any history of metastases or radiation therapy in the cervical-facial region [3,4].

Bisphosphonates (BF), drugs with antiresorptive properties, significantly reduce fracture risk in patients with benign bone disease and have demonstrated effectiveness in addressing skeletal-related events (SREs) in individuals with bone metastases (BM) [8]. Bisphosphonates are stable synthetic derivatives of pyrophosphate, a chemical compound characterized by the presence of two phosphate groups linked to a carbon atom by esterification. Similar to pyrophosphate, their natural analog, bisphosphonates have a very high affinity for bone minerals since they establish bonds with hydroxyapatite crystals [9,10]. By inhibiting the decomposition of hydroxyapatite, bisphosphonates can effectively suppress bone resorption, which makes them effective in treating bone metastasis in cancers [11,12,13]. Bisphosphonates act on osteoclasts by inhibiting the enzyme farnesyl-pyrophosphate-synthase, reducing differentiation and cellular activity, and increasing cellular apoptosis depending on drug concentration and half-life [14].

Zoledronic acid is a bisphosphonate administered intravenously (IV) to cancer patients, usually at a dosage of 4 mg at 4-week intervals for the purpose of preventing bone metastases [15,16]. It has a 100–1000 times greater effect than pamidronate [17]. The incidence of MRONJ in individuals diagnosed with cancer who received intravenous zoledronic acid for the prevention and control of bone metastases varies between 0 and 18%, with a cumulative risk below 5% [4]. This large variation is explained by different durations of follow-up between 1 and 10 years, in various studies [4]. The risk of developing MRONJ in a cancer patient who received intravenous zoledronic acid is 2–10 times higher than in a patient who received a placebo [4].

The main risk factor in the occurrence of MRONJ with zoledronic acid is represented by the treatment duration [4]. The risk of MRONJ in cancer patients who received intravenous zoledronic acid to prevent bone metastases was 0.5% after one year of administration, 1% after two years of administration, and 1.3% after three years of administration, being lower than that for denosumab [18]. However, in a review published in 2020, Limones et al. showed that the risk was higher, reaching 1.6% after one year, 2.1% after two years, and 2.3% after three years [19]. In a more recent review, the risk of MRONJ in cancer patients after taking zoledronic acid was even higher, ranging between 1.6 and 4% after two years of treatment and 3.8 and 18 % after more than two years of treatment [20]. In general, it has been observed that MRONJ appeared after a period of approximately two years of monthly administration of zoledronic acid, being correlated with the administration of zoledronic acid at intervals of less than five weeks [21].

The local factors that contribute to the occurrence of MRONJ are tooth extraction in a large percentage (70%) and mandibular localization in 75% [22].

According to the findings of a systematic literature review, the factors contributing to systemic risk for medication-related osteonecrosis of the jaw are age and gender [23,24,25], as well as cancer treatments (including chemotherapy, novel molecules, and corticotherapy) [24,26,27,28,29,30,31] and comorbidities such as hypertension, anemia, ischemic heart diseases, diabetes mellitus, dementia, and renal failure [26,32,33,34], and smoking [35]. The most frequently reported risk factors were chemotherapy, corticosteroid treatment, and smoking [26]. Healing from MRONJ takes longer in patients with diabetes and those treated with corticosteroids [33]. The occurrence of MRONJ has been observed with greater frequency in the elderly population as compared to younger individuals. Furthermore, this phenomenon has been reported to occur more frequently in patients receiving intravenous (IV) bisphosphonates as opposed to oral medications, particularly at higher dosages, especially when utilizing zoledronic acid. Additionally, a higher incidence of MRONJ has been noted in cancer patients undergoing chemotherapy and corticosteroid therapy [24].

The objective of the current study was to determine the systemic and local risk factors for MRONJ in a group of cancer patients treated with zoledronic acid for bone metastases and to establish the risk for MRONJ through a binomial regression analysis.

## 2. Materials and Methods

### 2.1. Study Design

The retrospective study used databases from two hospitals, County Clinical Emergency Hospital of Craiova and County Clinical Emergency Hospital of Constanta, and a cancer ambulatory treating center, Oncolab Craiova, all from Romania. The database contains information on patients’ demographic data (age, sex, residency, environment), cancer diagnosis and treatment, comorbidities, complications, inpatient and outpatient care in hospital, and ambulatory services. Data from the study were collected from March 2019 to December 2022. The study was approved by the Ethics Committee of the University of Medicine and Pharmacy of Craiova, no. 59/22.03.2019.

### 2.2. Patients

Patients included in the study were cancer patients treated with bisphosphonates approved in Romania for bone metastases from June 2018 to June 2022 in two geographical regions from Romania, South-West Oltenia, and South-East Dobrogea. The inclusion criteria were the following: patients previously diagnosed with various types of neoplasms and treated with 4 mg IV zoledronic acid administered once a month. The exclusion criteria were patients younger than 20 years, patients with only oral bisphosphonate treatment, patients treated with radiotherapy in the maxillo-facial area, patients treated with bisphosphonates for osteoporosis, and patients with oral cancers. Data analysis was performed between January 2021 and October 2022 and the results are in compliance with STROBE guidelines [36].

All participants in this study signed an informed consent form prior to their medical admission for treatment.

### 2.3. Outcome

The retrospective study compared risk factors encountered in cancer patients with MRONJ (the study group) to risk factors in cancer patients without MRONJ (control group).

The criteria used to define MRONJ published by AAOMS in a position paper from 2022 [4] were as follows:

1. Current or previous treatment based on antiresorptive or antiangiogenic agents;
2. The presence of exposed bone or an intraoral or extraoral fistula in the maxillofacial region that has persisted for more than eight weeks;
3. Patients with no history of radiotherapy to the jaws or obvious metastatic disease of the jaws.

Patients diagnosed with MRONJ were identified according to the above-mentioned criteria as established by position papers of AAOMS in 2014 and 2022 [3,4] and to the diagnostic codes and data from medical charts pertaining to surgical treatment of MRONJ performed in the two oral and maxillofacial surgery clinics from the two aforementioned geographical regions of Romania: the Oral and Maxillofacial Surgery Clinic of the University of Medicine and Pharmacy of Craiova, and Oral and Maxillofacial Surgery Clinic of the “Ovidius” University of Constanta, from Constanta. The surgical procedures employed to manage MRONJ comprised bone curettage involving sequestrectomy or jaw resection, with the objective of achieving a clinically viable bone.

The data collected pertaining to the enrolled patients comprised the interval between the first administration of bisphosphonate to the end of the observation period or to the culmination of the study (June 2022), whichever came first.

The sample size was computed using G*Power 3.1.9.7, Heinrich Heine University Düsseldorf, Germany. There were several parameters considered: a significance level α of 0.05, a power 1-β equal to 0.85, and a medium effect size value of 0.3. Consequently, the analysis led to a minimum enrolment of 160 patients for the study.

### 2.4. Data Acquisition

In the study, demographic data, comorbidities, and oncological data were extracted from the clinical records for each patient individually. The demographic data included the medical center, gender, age, residency, and smoking status. Comorbidities were assessed in terms of the presence or absence of bone metastasis, cardiovascular diseases, hypertension, diabetes mellitus, obesity, anemia, and renal diseases. With regard to oncological data, the primary diagnosis of the underlying disease was determined, along with the associated treatment, which may have involved chemotherapy, endocrine therapy, immunotherapy, radiotherapy, or corticotherapy. Furthermore, specific BF-related data such as the administration of zoledronic acid or other resorptive treatments, the type of administration, the duration of BF administration, and the presence or absence of osteonecrosis were recorded.

The data collected for all the subjects with MRONJ included the following: MRONJ detailed location (upper or lower jaw, or both), stage (according to the American Association of Oral and Maxillofacial Surgery (AAOMS) [3,4], the trigger factor (periodontal disease, periapical lesion, or extraction), subsequent treatment (bone resection, sequestrectomy, and curettage), and the presence of denudated bone or hypoesthesia.

### 2.5. Statistical Analysis

The data collected from the patients’ medical charts were initially processed using Microsoft Excel 365 (San Francisco, CA, USA). Consequently, this led to a basic distribution of the study group into subgroups. Continuous variables were presented as mean ± standard deviation and were compared using Kendall’s tau-b or Mann–Whitney U test for non-gaussian distributions. The categorical variables were expressed as numerical values and percentages, and their association was evaluated with either the Chi-square test or the Fisher Exact test. All statistical tests were employed using Statistical Package for Social Sciences (SPSS), version 20 (IBM Corp., Armonk, NY, USA). The acquired information was incorporated into a binomial logistic regression model to assess the likelihood of developing osteonecrosis in relation to the following variables: gender, age, smoking status, previous or current chemotherapy, endocrine therapy or cortico-therapy, duration of BF treatment, and smoking status. Linearity of the continuous variables with respect to the logit of the dependent variable was assessed via the Box–Tidwell (1962) procedure. A Bonferroni correction was applied using all thirteen terms in the model resulting in statistical significance being accepted when *p* < 0.003846. The α threshold was set to 5%, and the value *p* < 0.05 was considered statistically significant.

## 3. Results

### 3.1. Patients’ Characteristics

**Figure 1:**
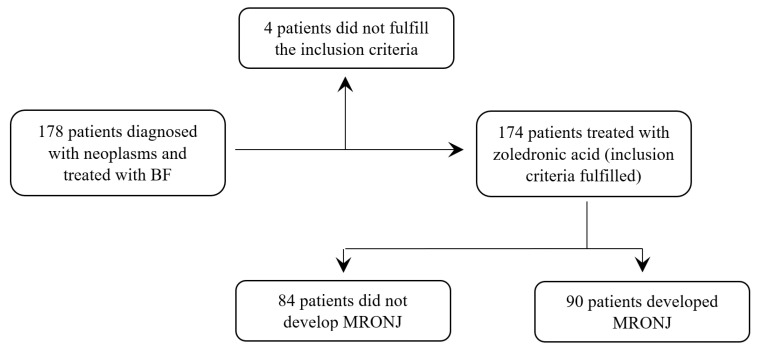
Study design and inclusion/exclusion criteria (BF—Bisphosphonates; MRONJ—Medication-Related Osteonecrosis of the Jaw).

The initial study group comprised 178 patients (Figure 1).

After the exclusion criteria were applied, in the study group remained 174 patients (Table 1), 109 females and 65 males, aged between 22 and 84 years old, with an overall average age of 64.6 ± 10.7, as the group included mostly elderly patients.

More than half of the patients were from Constanta (101 patients, 58.1%), while 73 patients (41.9%) were from Craiova. Patients were distributed in age groups, expressed as the following: 22–54 (young adults), 55–64 (mature adults), 65–71 (young old), and 72–84 years old (old old) (Table S1). For our study group, the most frequent comorbidities were cardiovascular diseases in 81 patients (46.5%, specific for elderly participants), hypertension in 64 patients (36.8%), nutritional diseases in 56 patients (32.2%), diabetes mellitus in 18 patients (10.4%), obesity in 16 patients (9.2%), renal diseases in 31 patients (17.8%), and anemia in 18 patients (10.4%). Comorbidities’ distribution by age group and gender is presented in Table 2.

All 174 patients were previously diagnosed with neoplasms, of which breast and prostate cancers were predominant in our study group. Overall, 82 females had breast cancer (47.2%), 46 males had prostate cancer (26.4%), while the rest of the 46 patients had other neoplasms: pulmonary, myeloma, genital, digestive, renal, cerebral, bladder, spinal cord, pharynx, or thyroid. Bone metastases were diagnosed in 154 patients (88.5%, almost two-thirds being females, mostly encountered for ages above 65 years old). Neoplasms’ general distribution is presented in Table S2. From the entire study group, more than three-quarters of patients received chemotherapy (alone or in association with molecular-targeted therapy) (86.8%, 76 females with breast neoplasm, 34 males with prostate neoplasm, and 41 patients with other neoplasms) (Appendix A), while 39.1% underwent radiotherapy (29 females with breast neoplasm, 20 males with prostate neoplasm, and 19 patients with other neoplasms), 20.7% received endocrine therapy (12 females with breast cancer, and 24 males with prostate cancer), 5.8% corticosteroids (6 females with breast cancer, 2 males with prostate cancer, and 2 patients with pulmonary neoplasms, 1 female and 1 male), and only 1.2% underwent immunotherapy (2 females with renal and pulmonary neoplasms). Patients’ distribution by treatment is presented in Table 3. More than 50% of patients underwent one single therapy type, 36.2% (63 patients) received two types of therapy, and 6.9% (12 patients, 6 females with breast neoplasm and 6 males with prostate neoplasm) experimented with three different types of neoplasm therapy, while 2 patients (1.2%, both females with breast neoplasm) underwent 4 different types of therapy.

Among the cohort of patients who received chemotherapy, 133 had bone metastasis, 68 had cardiovascular diseases, from which 52 had hypertension, 49 had nutritional diseases (17 patients had diabetes mellitus, 16 were obese), 24 had renal diseases, and 17 were anemic. Endocrine therapy was recommended mostly for more than half of all males with prostate cancer and with few other comorbidities, the most common being cardiovascular and renal diseases. Patients with radiotherapy had a similar distribution regarding the neoplasm type; however, in Craiova, this type of therapy was mostly recommended for females with breast cancer. All 10 patients who underwent corticotherapy were from Craiova, mostly females aged less than 64 years old, with very few comorbidities except bone metastasis (5 patients with nutritional diseases, 2 with renal diseases, and 1 female with cardiovascular diseases). Immunotherapy was used only in two cases, one in Craiova, and one in Constanta, for elderly females, both with cardiovascular diseases and other comorbidities.

### 3.2. Bisphosphonate Treatment

The entire cohort within the study was subjected to the therapeutic intervention of BF-zoledronic acid, administered intravenously every month with a dosage of 4 mg. Regarding the duration of treatment, 29.9% had less than 12 months of BF treatment, 34.5% had between 12 and 24 months of treatment, 19.5% had between 2 and 3 years of treatment, while 16.1% received BF treatment for more than 3 years. The average treatment duration for each age group is presented in Table 4.

### 3.3. MRONJ Distribution Analysis

Approximately half of all patients (90 patients, 51.7%) who developed osteonecrosis of the jaw had an average treatment duration with zoledronic acid of 29.0 ± 12.1 months; the rest of the 84 patients (48.3%) who did not develop MRONJ had an average treatment duration of 19.6 ± 17.9 months). Of the 90 patients with MRONJ, 53 were from Craiova (58.9%), and only 37 were from Constanta (41.1%). A Chi-square test for homogeneity was conducted between the medical center and MRONJ development. There was a statistically significant difference regarding the proportion of patients with MRONJ from Craiova compared to patients with MRONJ from Constanta, χ^2^(1) = 21.9, *p* < 0.0005.

Kendall’s tau-b correlation was run to determine the relationship between age and MRONJ presence amongst all 174 participants. There was a moderate, positive association between age and osteonecrosis development (mean age 65.5 ± 9.5 for patients with MRONJ, and 63.7 ± 11.9 for patients without MRONJ), which was not statistically significant, τ_b_ = 0.055, *p* = 0.382. In addition, a Chi-square test of homogeneity was conducted between the two groups with and without MRONJ in relation to the patients’ age group, with an adequate sample size established according to Cochran (1954). There were no differences in proportions between the four age groups, χ^2^(3) = 4.985, *p* = 0.173. Observed frequencies and percentages of age groups for each group are defined in Table 5. There were no statistical differences in developing MRONJ regarding gender, residency, or smoking status.

Approximately half of all females with breast cancer developed jaw osteonecrosis (52.4%); however, this cancer type does not seem to be a risk factor for developing MRONJ in females (χ^2^(1) = 0.079, *p* = 0.778). Similar results were obtained for males and prostate cancer, with 52.2% of males having jaw osteonecrosis (χ^2^(1) = 0.545, *p* = 0.460). The overall analysis of neoplasm types (breast, prostate, and other neoplasms) did not reveal any statistically significant differences regarding MRONJ development (χ^2^(2) = 0.075, *p* = 0.963).

For this study group, two types of neoplasm therapies were associated with MRONJ: chemotherapy (as 94.4% of patients developed MRONJ), and corticotherapy. In this case, all treated patients developed MRONJ. Endocrine therapy seems a protective factor.

Of the 90 patients with MRONJ, 71 patients (representing 78.9%) had stage 2 MRONJ, and 19 patients (21.1%) had stage 3 MRONJ (Table 6). A chi-square goodness-of-fit test was conducted to determine whether an equal number of patients for both MRONJ stages were represented in the study. The minimum expected frequency was 45. The chi-square goodness-of-fit test indicated that the number of patients with stages 2 and 3 was statistically significantly different (χ^2^(1) = 30.044, *p* < 0.0005), with more than three-quarters of patients having stage 2 MRONJ.

Kendall’s tau-b correlation was run to determine the relationship between age and MRONJ stage amongst all 90 participants. There was a very weak, negative association between age and stage (mean age 65.9 ± 8.8 years old for patients with stage 2, and 64.4 ± 12.0 years old for patients with stage 3), which was not statistically significant, τ_b_ = −0.025, *p* = 0.778. Similarly, there were no statistical differences in the MRONJ stage regarding residency, gender, smoking status, or the medical center.

**Figure 2:**
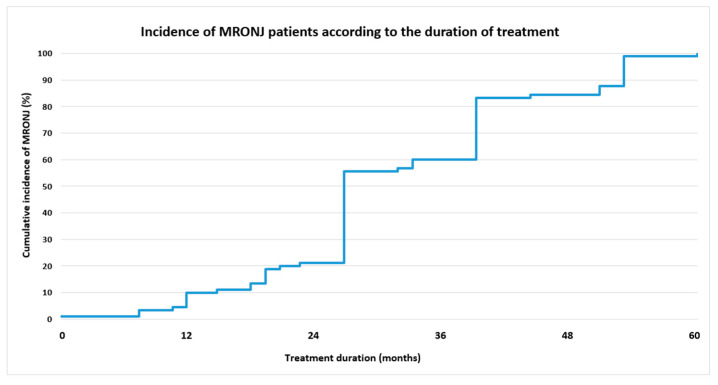
Cumulative incidence of MRONJ patients according to the duration of BF treatment.

Bisphosphonates treatment duration was recorded in months, and the following categories were identified: 1–12 months (52 patients, 29.9% from the entire study group), 13–24 months (60 patients, 34.5% from the entire study group), 25–36 months (34 patients, 19.5% from the entire study group), and >36 months (28 patients, 16.1% from the entire study group) (Table 7). The cumulative incidence of MRONJ patients according to the duration of BF treatment is presented in Figure 2.

A Mann–Whitney U test was run to determine if there were differences in treatment duration between MRONJ and non-MRONJ groups. Distributions of duration were not similar, as assessed by visual inspection. Median treatment duration was statistically significantly higher for the MRONJ group (24 months) than for the non-MRONJ group (12 months), U = 5638, z = 5.620, *p* < 0.0005. Treatment duration was not related to the MRONJ stage (U = 704, z = 0.300, *p* = 0.764).

The following trigger factors were identified in MRONJ development: tooth extraction (51 patients, 56.7% of all MRONJ patients), periapical disease (26 patients, 28.9%), and periodontal disease (13 patients, 14.4%). A chi-square goodness-of-fit test was conducted to determine whether an equal number of patients for the three trigger factors were represented in the study. The minimum expected frequency was 45. The chi-square goodness-of-fit test indicated that the number of patients with various trigger factors was statistically significantly different (χ^2^(2) = 24.867, *p* < 0.0005), with more than half of patients having extraction as a trigger factor.

For patients from the first age group, the main trigger factor was periodontal disease (with 50% from this group), compared to the other age groups, where it was the least representative factor. Periapical disease was predominant for groups 55–64 and 72–84 years old, while extraction was the main factor for patients with ages above 54 years old. Overall, the differences between age groups regarding the trigger factor were statistically significant, χ^2^(6) = 15.752, *p* = 0.015.

Of the 90 patients with MRONJ, 58 patients (representing 64.4%) developed MRONJ in the lower jaw, compared to only 31.2% (28 patients) in the upper jaw, while 4 patients (4.4%) presented osteonecrosis in both jaws. A chi-square goodness-of-fit test indicated that the number of patients for each location was statistically significantly different (χ^2^(2) = 48.800, *p* < 0.005), with approximately two-thirds of the patients presenting mandibular osteonecrosis. Age group analysis revealed significant differences between patients with ages below 55 years old, with predominant upper jaw MRONJ (60% from the entire group), and the other three age groups, with predominant lower jaw MRONJ (73.1% for group 55–64, 51.9% for group 65–71, and 81.5% for group 72–84 years old), χ^2^(6) = 13.348, *p* = 0.038.

The presence of denudated bone was recorded for 87 patients, representing 96.7%, compared to only 3 patients without denudated bone (3.3%). According to the result of the chi-square goodness-of-fit test, the difference was statistically significant (χ^2^(1) = 78.400, *p* < 0.0005). Similar results were obtained regarding hypoesthesia, as it was identified for only 16 patients (17.8%), compared to 74 patients (82.2%), and this difference was statistically significant (χ^2^(1) = 37.378, *p* < 0.0005).

Surgical treatment for patients with osteonecrosis of the jaw was represented by sequestrectomy (64 patients, 71.1% from the entire study group), resection (21 patients, 23.3% from the entire study group), or curettage (7 patients, 7.8% from the entire study group). Among this study lot, sequestrectomy was the election surgical procedure for more than two-thirds of patients; for statistical purposes, these patients have been grouped with patients with curettage, with the differences between the number of patients with and without these procedures being statistically significant (χ^2^(1) = 4.003, *p* < 0. 0.045).

Resection was performed for less than a quarter of patients, reflecting statistically significant differences between the number of patients with and without resection (χ^2^(1) = 25.600, *p* < 0.0005).

The majority of MRONJ patients underwent one surgical procedure (76 patients, 84.4%), eight patients suffered a relapse and underwent a second surgical intervention (8.9%), while six patients (6.7%) were not surgically treated. With a minimum expected frequency of 30 for each category, a chi-square goodness-of-fit test reflected statistically significant differences between the number of patients with one or more surgical procedures (χ^2^(2) = 77.400, *p* < 0.0005), with around three-quarters of patients being the subject of a single surgical procedure.

Several common comorbidities have been analyzed for this study group. Following a Chi-Square test, only cardiovascular diseases (mostly hypertension) may be considered a risk factor for developing MRONJ (Table 6), with χ^2^(1) = 9.443 and *p* = 0.002. The analysis of the MRONJ stage in relation to comorbidities revealed similar results.

### 3.4. Binomial Logistic Regression

From the binomial logistic regression performed to ascertain the effects of gender, age, smoking status, treatment duration with zoledronic acid, chemotherapy, radiotherapy, endocrine therapy, presence of DM, obesity, and hypertension, on the likelihood that patients develop osteonecrosis of the jaw, the results indicate that all continuous independent variables were found to be linearly related to the logit of the dependent variable (Box–Tidwell procedure). There were three standardized residuals with values around three standard deviations, which were kept in the analysis. The logistic regression model was statistically significant, χ^2^(10) = 62.406, *p* < 0.0005. The model explained 40.2% of the variance in osteonecrosis development and correctly classified 76.4% of cases. Sensitivity was 81.1%, specificity was 71.4%, positive predictive value was 75.26%, and negative predictive value was 77.92%. The area under the ROC curve was 0.824 (95% CI, 0.761 to 0.886), which is an excellent level of discrimination. Of the ten predictor variables, only five were statistically significant: duration of treatment, chemotherapy, endocrine therapy, obesity, and hypertension (Table 8).

Cancer patients undergoing chemotherapy had 7.53 times higher odds to develop osteonecrosis of the jaw, while endocrine therapy was associated with a reduced frequency of MRONJ. Patients with hypertension had 3.79 times higher odds to develop osteonecrosis of the jaw than the patients with normal blood pressure values, while obesity was associated with a reduction in the likelihood of developing osteonecrosis. Longer zoledronic acid treatment duration was associated with an increased risk for MRONJ.

## 4. Discussion

Since MRONJ is a complication with a major negative impact on the quality of life of cancer patients [7,37,38,39], a risk factors evaluation for each patient before the treatment initiation with bisphosphonates or other drugs to control bone metastases is necessary. In order to enable customized therapy for both bone metastases and oral diseases that may cause MRONJ lesions, it is advisable to establish an individual risk profile of the patient [27]. Several studies have made correlations between systemic and local risk factors and MRONJ [27,28,32], and among them, the study by Marciano et al. [27] showed that by knowing the patient’s risk profile, risk stratification can be achieved, and plans can be made for performing elective dental procedures in safe conditions, thus preventing MRONJ.

Like the study by Marciano et al. conducted in an Italian population [27], the current study presents a descriptive statistical analysis of a part of a Romanian cancer population from two geographical regions who were treated with zoledronic acid, as well as the results of the binomial logistic regression analysis through which several risk factors were associated with the occurrence of MRONJ. As some researchers have pointed out [32], the geographical origin of the population studied is important since genetic and environmental factors could be different [40,41,42]. Our study groups belong to two different geographical regions of the country situated 450 kilometers apart, one with a sea-side opening (Dobrogea, with its municipality in Constanta, South-East Romania), and the other, in the plain area close to Bulgaria (Oltenia, with its municipality in Craiova, South-West Romania).

The incidence of MRONJ in zoledronic acid-treated cancer patients varies between 0.2% and 9.9%, reaching 15–20% in some case studies [27,38,43]. The present study aimed to explore the risk factors associated with MRONJ by studying two groups of patients almost equal in number treated with zoledronic acid, with the study group who developed MRONJ and the control group, without MRONJ. The present study described the association of MRONJ with various risk factors: demographic factors, cancer type and cancer treatment, co-morbidities, and duration of bisphosphonate treatment. MRONJ characteristic associations with risk factors were performed for the MRONJ stage, trigger factor, lesion location, denudated bone presence, hypoesthesia presence, and surgical treatment type.

The results of the binomial logistic regression performed on ten predictor variables (gender, age, smoking status, treatment duration, chemotherapy, radiotherapy, endocrine therapy, diabetes mellitus, obesity, and hypertension) pertaining to the likelihood of MRONJ occurrence, showed that only five were statistically significant, namely, chemotherapy, hypertension, duration of treatment, endocrine therapy, and obesity.

Romania is among the top 10 European countries with the highest mortality rates due to cancer [15,44]. The studied groups included patients residing in two geographical areas of the country with a high prevalence of cancer. The highest prevalence of cancer in Romania in 2019 was recorded in the S-W Oltenia region (of which Dolj county is a part) (3283.64/10,000) followed by the prevalence in the S-E region (Dobrogea) (3033.3/10,000) [15,45,46,47].

Although more than half of the patients were from the S-E Dobrogea region (101 patients, 58.0%), compared to 73 patients (42.0%) from the S-W Oltenia region, out of 90 patients with MRONJ, the majority (53, 58.9%) were from S-W Oltenia and only 37 were from S-E Dobrogea (41.1%) *p* < 0.05. This can be correlated with the fact that the highest prevalence of cancer in Romania in 2019 was recorded in the S-W Oltenia region (3283.64/10,000) followed by the prevalence in the S-E region (Dobrogea) (3033.3/10,000) [46,47], with most new cancer cases in 2019 being reported in the S-West Oltenia region, (367.8/10,000) [45,46,47]. In a recent study, a weak association was identified between geographic location and the development of MRONJ in patients with cancer [32].

The majority of the patients in the studied groups were women (over 62%), over 55 years old (over 82%), more from the region of S-E Dobrogea, Constanta (over 58%), and more from the urban environment (over 71%). Most of the population studied (over 73%) were diagnosed with breast or prostate cancer. Another study published in 2020 regarding the incidence of MRONJ in the period 2009–2018 in patients from Craiova (S-W Oltenia) treated with bisphosphonates showed that most patients with MRONJ were women; the age of the patients was higher in men compared to women and the origin was urban for most patients [48].

In a study conducted by Ishimaru et al., in 2021, middle-advanced age (65 to 74 years old) was correlated with the occurrence of MRONJ in cancer patients, especially in men [32]. Rodriguez-Archilla et al., in 2019, performed a review (meta-analysis) on studies from the PubMed database regarding predictive risk factors for the occurrence of MRONJ. In this meta-analysis, an examination of twenty-five studies regarding predictive risk factors of MRONJ revealed the identification of certain factors, such as advanced age and female sex, as posing increased risk [49].

Most of the patients in our study group were from an urban environment, which is consistent with another previous study [48]. Statistical data indicated that the incidence of cancer was significantly higher in the urban environment compared to the rural environment [15,45,46,47]. The addressability to the oncologist, expressed as hospitalized morbidity (patients who were hospitalized for cancer treatment), was lower in the rural areas than in the urban areas, being the lowest in the S-E and S-W Oltenia regions [45,46,47,50]. Most patients from the urban environment came from the region of S-E Dobrogea, Constanta (over 58%). Although almost three-quarters of patients from the study group (125 patients, representing 71.8%) lived in urban areas (with a male/female ratio of approximately one-third), and 49 patients (28.2%) lived in rural areas (with a similar gender distribution); residency did not have a significant risk association with MRONJ.

One identified risk factor that has been linked to a higher incidence of MRONJ is the type of cancer, namely, breast and prostate cancers [21,22]. Breast and prostate cancers are among the cancers with high mortality in Romania. Breast cancer has a mortality of 15.5/100,000 inhabitants and prostate cancer of 10.6/100,000 inhabitants [43]. In the current study, the majority (over 73%) of the patients in the group were diagnosed with breast or prostate cancer, with more women than men in the group. Breast cancer is the seventh leading cause of death in Romania, while prostate cancer ranks 10th among the leading causes of death in Romania, as shown by the World Health Rankings [51,52]. Breast cancer is characteristic of middle-aged women, while prostate cancer is characteristic of elderly men; the incidence gradually increasing until the last age group where it is at its maximum [45,46,47]. In our study, 54.9% of breast cancer occurred in women between 55 and 71 years of age, and 76.1% of prostate cancer occurred in men between 65 and 84 years, with a much higher frequency after 72 years (45.6%). Another retrospective pilot study conducted retrospectively from 2012 to 2017 revealed a substantial incidence of medication-related osteonecrosis of the jaw (MRONJ) in patients receiving intravenous (IV) zoledronic acid as a treatment for bone metastases associated with breast or prostate cancer [50].

In a review regarding MRONJ, Anastasilakis et al. [8] showed that the risk for MRONJ was much higher in patients with advanced malignancies compared to those with benign bone diseases, due to higher doses and the more frequent administration of antiresorptive agents in people with compromised general health, together with the concomitant administration of other drugs that predispose to MRONJ. In the study by Hata et al. [53], the cumulative incidence of MRONJ in breast cancer, prostate cancer, and multiple myeloma was found to be related to the frequency of antiresorptive drugs and the duration of treatment. Patients with cancers with median survival times of less than 10 months did not develop MRONJ. In renal cancer, the cumulative incidence of MRONJ increased early, with a median survival time of 12 months.

Overall, these studies emphasize the importance of dental prophylaxis and maintenance of good oral hygiene for the prevention of MRONJ in patients treated with bisphosphonates and denosumab and highlight important factors in the risk of development and re-occurrence of this condition [8,54]. A study that followed the cumulative incidence of MRONJ after 3 years, in patients with bone metastases treated with zoledronic acid, showed that the type of cancer, oral health, and frequency of antiresorptive use were associated with the risk of MRONJ [21]. Moreover, patients undergoing treatment with denosumab or zoledronic acid for bone metastases from breast, multiple myeloma, or prostate cancers have a higher risk than those with lung cancer of developing MRONJ [27].

Several studies have suggested that comorbidities may constitute potential risk factors for the development of MRONJ [32,34]. Comorbidities affect patients older than 50 years, with most of the studied patients having at least one comorbidity. Cardiovascular disease was found especially in the two groups of elderly patients, almost a third of them, and hypertension had a similar distribution. Binomial regression analysis performed in the present study revealed that patients with hypertension had 3.79 times higher odds of developing medication-related osteonecrosis of the jaw than the patients with normal blood pressure values, while obesity was associated with a reduced risk of developing osteonecrosis. The first two causes of death in Romania are represented by coronary heart disease and stroke, a complication of hypertension [51]. Occupying fifth place among European countries in terms of high cardiovascular risk, according to ESC statistics, Romania has hypertension as the main risk factor (39.1%), along with hypercholesterolemia (39.1%), followed by smoking (26.7%) and obesity (21.3%) [16,54].

In Romania, campaigns have been carried out with the aim of involving authorities and the media in increasing awareness regarding diabetes prevention and control [55]. In the present study, nutritional diseases (diabetes, obesity) were distributed almost equally in the last three age groups. Consequently, the patients were affected by these diseases starting at the age of 55 (after 50). In Romania, the prevalence of diabetes is estimated at 11.6% in the population aged between 20 and 79 years. Newly reported cases represent 20.7% [55]. Patients with type 2 diabetes mellitus (T2DM) are at higher risk of cardiovascular disease, and age strongly predicts cardiovascular complications [56].

In the study conducted by Ishimaru et al., dementia and renal diseases related to renal cancer, which was the most common form of cancer associated with MRONJ in the patients studied, were identified as significant comorbidities [32]. In the current study, kidney diseases were found in the last two age groups of patients, thus affecting only 31 of the 174 patients. According to the latest statistical reports, the prevalence of kidney diseases correlates with the prevalence of hypertension [57].

Anemia is found in all four groups of patients (this is the anemia that the patient had at the time of the first visit, because is known that chemotherapy treatment produces anemia) [58].

Several studies have identified chemotherapy as an important risk factor for MRONJ [24,26,27,28,29,30,31,59]. Chemotherapy is the treatment of choice for neoplasms in Romania, and of the total number of cancer patients studied, 86.78% received this treatment. From the results of binomial regression analysis, in the present study, patients with chemotherapy had 7.53 times higher odds of developing MRONJ, while endocrine therapy was associated with a reduced frequency of MRONJ. In the study by Kawahara et al., chemotherapy was associated with MRONJ at a rate of 39.7%, followed by corticotherapy (24.6%) [59].

Among the other cancer therapies, radiotherapy was used more frequently, while endocrine therapy was used less often as it is reserved especially for less serious cases of breast and prostate cancer. Immunotherapy was very rarely encountered [16,51,52,60,61,62].

In the binomial regression analysis from the present study, a longer zoledronic acid treatment duration was associated with an increased risk for MRONJ. The average duration of treatment with bisphosphonates was longer in Craiova than in Constanta, longer in patients with prostate cancer, followed by breast cancer, and then by the other types of cancer. MRONJ appeared in a significantly higher percentage of patients from Craiova, who had a longer duration of treatment with BF (2 months longer on average). The duration of treatment with bisphosphonates is presented as the main risk factor for MRONJ in several studies [27,39,61,62]. In the present study, the median treatment duration was statistically significantly higher for the MRONJ group (24 months) than for the non-MRONJ group (12 months), *p* < 0.05. A duration of more than 12 months (an average of 18 months, meaning one year and a half) doubles the risk of MRONJ development, while a duration of more than 24 months (an average of 32 months, meaning two years and a half) triples it. The relationship between the risk of MRONJ development and the duration of treatment with antiresorptive agents in cancer patients was also analyzed in other studies [38,63,64], reporting a median duration of treatment of 17.5 months [63] or 19 months [64].

Treatment duration was not related to the MRONJ stage. In other studies, the average duration of treatment with BF was between 12 and 55 months [61]. According to several studies, the duration of treatment with bisphosphonates of more than 10 doses was a significant risk factor for the occurrence of MRONJ in patients treated with zoledronic acid [27,39,62]. In the current study, the average duration of treatment with bisphosphonates was the highest in the 55–64 age group, followed by the older age groups (almost equal values—approx. 2 years of treatment), with the young age group having the shortest treatment period, under one and a half years. The duration of treatment with zoledronic acid is influenced by the bone metastases, the evolution of the underlying disease, and the duration of survival/healing of the patients [27,39,62]. Several studies have shown that patients treated with zoledronic acid or denosumab for more than 18 months have an increased risk of MRONJ recurrence and that the number of doses of zoledronic acid or denosumab administered, exposure to new chemotherapeutic compounds, and the type of cancer, are important factors in the risk of developing MRONJ [27,38,62].

According to Kemp et al., among the risk factors for medication-related osteonecrosis of the jaw in oncological patients treated with zoledronic acid is the monthly administration rate, as well as the lack of dental control, surgical procedures (especially tooth extraction), and smoking. In Kemp’s study, the localization of MRONJ in the upper jaw predominates; unlike in our study, where most patients had MRONJ in the lower jaw [39].

Studies have also been carried out regarding the association of risk factors with the characteristics of MRONJ, such as the stage of MRONJ, the trigger factors, the location, the presence of exposed bone, the presence of hypoesthesia, and the type of surgical treatment.

In the present study, the most important trigger factor for MRONJ was tooth extraction, followed by periapical disease and periodontal disease. More than half of the patients had extraction as a trigger factor (*p* < 0.0005). Age groups were correlated with the trigger factor, and the differences between age groups regarding the trigger factor were statistically significant (*p* = 0.015): extraction was the main risk factor for patients with ages above 54 years old, periapical disease was predominant for groups 55–64 and 72–84 years old, and periodontal disease was the main trigger factor for patients from the first age group.

There were also other studies that reported dental extraction as a trigger factor of MRONJ in a proportion of 61.7–75% [39,59]. The incidence in patients diagnosed with MRONJ after dental extractions was 2.28 per 100,000 people/year [32].

According to the study conducted by Wick et al., local inflammation is considered the main trigger of MRONJ, while the suggestions of Aguirre et al. indicate that oral risk factors lead to osteocyte necrosis in MRONJ through TNFα/TNFR1 signaling and enhance the inflammatory response [65,66].

Another risk factor for MRONJ was the localization of the lower jaw lesion. Thus, in our study, 64.4% of the patients presented MRONJ in the mandible compared to only 31.1% in the maxilla. A small number (4.4%) of patients had osteonecrosis in both jaws. Similar rates regarding the localization of MRONJ were also recorded in the study by Kawahara et al. in 2021 [59]. Some studies have shown that there were even greater differences between the two jaws in terms of MRONJ localization: the lower jaw being affected in over 71% of patients compared to less than 22.5% in the upper jaw [61,67]. An explanation of the recorded differences could be due to less vascularity and a thinner mucosa in the mandible compared to the maxilla [68]. The most common location of the MRONJ area in the mandible would be the mandibular ramus, followed by the mandibular body and mandibular symphysis [69,70,71]. However, there are studies that have shown that the upper jaw was more affected by osteonecrosis than the mandible, but the number of reported patients was much lower [39].

In our study, the most frequent MRONJ localization was in the posterior area both in the maxilla and in the mandible, without any statistical significance. The same was observed in the study by Feng et al. [61].

A correlation between the location of osteonecrosis and the age of the patients was noticed. Mandible osteonecrosis was encountered for patients over 55 years old while maxillary osteonecrosis was predominant (60%) in patients with ages below 55 years old.

One of the clinical characteristics of MRONJ with a significant statistical value was the exposed bone, in a proportion of 96.67%. Hypoesthesia was present in only 17.78% of MRONJ patients. Other studies showed that focal and diffuse bone sclerosis and the occurrence of bone sequestrations could be observed more frequently in patients with exposed bone, compared to patients without exposed bone [33].

Most of the patients analyzed in this study (78.90%) had MRONJ stage 2, and only 10% were diagnosed with MRONJ stage 3. The MRONJ stage determines the choice of treatment method. Although in 2014, the AAOMS considered that surgical treatment is not recommended for patients with stage 1 and 2 MRONJ and should be limited to patients with stage 3 lesions or stage 2 lesions unresponsive to non-surgical treatment; other studies suggest that the surgical removal of necrotic bone may be an effective treatment for all stages of MRONJ [13,72]. Another study suggests that only patients in stages 2 and 3 of MRONJ should be admitted for surgical treatment [61].

A surgical approach may be considered for any necrotic bone exposed when conservative treatment has failed. A conservative surgical approach can be achieved through sequestrectomy associated with the administration of antibiotics and rinsing with antiseptic solutions [6,71]. The present study showed that sequestrectomy was the surgical procedure used in more than two-thirds of patients, with the differences between the number of patients with and without this procedure being statistically significant. Conservative surgery can be combined with other treatments such as ozone therapy or local application of PRF (plasma-rich fibrin). Studies have shown that ozone therapy has a lower percentage of positive results compared with local PRF applications [67].

Although the treatment of patients with an established diagnosis of drug-induced osteonecrosis of the jaw (MRONJ) should be approached with a pragmatic multidisciplinary treatment, prioritizing the patient’s quality of life and the management of their skeletal disease, sometimes a complete resection of the necrotic bone (i.e., a surgical intervention—extensive way) is necessary to achieve complete healing [13,72].

More recent studies have shown that stages 2 and 3 of MRONJ could be treated both surgically with or without adjuvant therapies, as well as conservatively [67,69,73,74,75,76]. Conservative treatment is the main treatment method of MRONJ, and although it does not always completely heal the lesion, it can provide long-term relief of symptoms [73]. Stages 1 and 2 of MRONJ can be completely cured by surgical treatment, while stage 3 is partially cured with an MRONJ stage regression according to the AAOMS [74].

In the present study, resection was performed for less than a quarter of patients, reflecting statistically significant differences between the number of patients with and without resection (*p* < 0.05). More than three-quarters of patients underwent only one surgical procedure, while 8.9% of patients underwent two different surgical procedures, and 6.70% of patients were not surgically treated (*p* < 0.05). Although in stage 3 MRONJ, the treatment of choice was most often surgical, the general rate of complications was high, with relapses being recorded [71]. Surgical treatment of MRONJ in the jaw can be an effective method, recording a rate of healing increase according to the study by Okuyama et al. [75].

The strengths of this study are reflected by a sufficient follow-up time (of 4 years) for all 174 patients residing in the two geographical regions, allowing the analysis of possible risk factors regarding demographic factors, cancer type and cancer treatment, co-morbidities, period duration of bisphosphonate treatment use, and also the MRONJ stage, trigger factor, overall lesion location, denudated bone presence, hypoesthesia presence, and surgical treatment type. Binomial regression analysis performed for ten predictor variables indicated the statistically significant factors: chemotherapy, hypertension, duration of treatment, endocrine therapy, and obesity. Chemotherapy increased 7.53 times the odds of developing osteonecrosis of the jaw of the cancer patients, while endocrine therapy was associated with a reduction in the likelihood of developing osteonecrosis. Hypertension increased 3.79 times the odds of developing MRONJ in the cancer patients, while obesity was associated with a reduction in the likelihood of developing osteonecrosis. Increasing treatment duration was associated with an increased likelihood of developing MRONJ (*p* < 0.005).

The limitations concern the lack of data regarding the oral health of the subjects before BF treatment initiation and regular oral monitoring during the treatment.

## 5. Conclusions

Risk factors identified for MRONJ correlated with zoledronic acid were chemotherapy, hypertension, and duration of treatment. Cancer patients with chemotherapy or hypertension had higher odds of developing MRONJ, while endocrine therapy and obesity were associated with a reduced frequency of MRONJ. Increased treatment duration with zoledronic acid was associated with an increased risk of developing MRONJ.

## Figures and Tables

**Table 1:** Participants’ characteristics extracted from the medical charts.

Category	Parameter	Value	N (%)/Mean ± SD/Interval
Demographic factors	Gender	Female	109 (62.6%)
		Male	65 (37.4%)
	Age (years old)	22–54	30 (17.4%)/46.9 ± 6.5
		55–64	46 (26.3%)/60.3 ± 2.7
		65–71	50 (28.7%)/67.8 ± 2.1
		72–84	48 (27.6%)/76.6 ± 3.7
	Medical center	Craiova	73 (41.9%)
		Constanta	101 (58.1%)
	Residency	Urban	125 (71.8%)
		Rural	49 (28.2%)
	Smoking status	No	100 (57.5%)
		Yes	74 (42.5%)
Cancer type	Primary diagnostic (neoplasm)	Breast	82 (47.2%)
		Prostate	46 (26.4%)
		Other neoplasms	46 (26.4%)
		Pulmonary	14 (30.4%)
		Myeloma	9 (19.6%)
		Genital	7 (15.2%)
		Digestive	6 (13.0%)
		Renal	5 (10.8%)
		Cerebral	1 (2.2%)
		Bladder	1 (2.2%)
		Spinal cord	1 (2.2%)
		Pharynx	1 (2.2%)
		Thyroid	1 (2.2%)
Metastasis	Bone metastasis	No	20 (11.5%)
		Yes	154 (88.5%)
Cancer therapy	Chemotherapy	No	23 (13.2%)
		Yes	151 (86.8%)
	Endocrine therapy	No	138 (79.3%)
		Yes	36 (20.7%)
	Immunotherapy	No	172 (98.8%)
		Yes	2 (1.2%)
	Radiotherapy	No	106 (60.9%)
		Yes	68 (39.1%)
	Corticotherapy	No	164 (94.2%)
		Yes	10 (5.8%)
BF	BF type	Zoledronic acid	174 (100%)
	BF	IV Zoledronic acid	169 (97.1%)
		IV Zoledronic acid administered after a treatment with oral BF	5 (2.9%)
	Treatment duration (months)	<12	52 (29.9%)/8.2 ± 3.2
		13–24	60 (34.5%)/21.0 ± 3.8
		25–36	34 (19.5%)/34.1 ± 3.1
		>36	28 (16.1%)/50.3 ± 12.1
Comorbidities	Cardiovascular diseases	No	93 (53.5%)
		Yes	81 (46.5%)
	Hypertension	No	110 (63.2%)
		Yes	64 (36.8%)
	Nutritional diseases	No	118 (67.8%)
		Yes	56 (32.2%)
	Diabetes mellitus	No	156 (89.6%)
		Yes	18 (10.4%)
	Obesity	No	158 (90.8%)
		Yes	16 (9.2%)
	Anemia	No	156 (89.6%)
		Yes	18 (10.4%)
	Renal diseases	No	143 (82.2%)
		Yes	31 (17.8%)

BF—Bisphosphonates. IV—intravenous.

**Table 2:** Distribution of patients by comorbidities, age groups, and gender.

Comorbidities	Total N	Age Groups/Gender—Number of Patients (%)							
		22–54		55–64		65–71		72–84	
		F	M	F	M	F	M	F	M
Cardiovasc. diseases	81	4 (4.9%)	4 (4.9%)	11 (13.6%)	7 (8.6%)	18 (22.2%)	10 (12.4%)	17 (21.0%)	10 (12.4%)
Hypertension	64	-	2 (3.1%)	10 (15.6%)	6 (9.4%)	15 (23.4%)	8 (12.5%)	14 (21.9%)	9 (14.1%)
Nutritional diseases	56	7 (12.5%)	2 (3.6%)	10 (17.9%)	5 (8.8%)	10 (17.9%)	7 (12.5%)	7 (12.5%)	8 (14.3%)
Diabetes mellitus	18	-	1 (5.6%)	2 (11.1%)	4 (22.2%)	2 (11.1%)	2 (11.1%)	4 (22.2%)	3 (16.7%)
Obesity	16	3 (18.7%)	-	3 (18.7%)	1 (6.3%)	5 (31.3%)	-	1 (6.3%)	3 (18.7%)
Renal diseases	31	1 (3.2%)	1 (3.2%)	3 (9.7%)	1 (3.2%)	1 (3.2%)	7 (22.6%)	6 (19.4%)	11 (35.5%)
Anemia	18	3 (16.7%)	1 (5.6%)	3 (16.7%)	-	1 (5.6%)	4 (22.2%)	4 (22.2%)	2 (11.0%)
Total		18	11	42	24	52	38	53	36

**Table 3:** Distribution of patients by therapy, age groups, and gender.

Therapy	Total N	Age Groups/Gender—Number of Patients (%)							
		22–54		55–64		65–71		72–84	
		F	M	F	M	F	M	F	M
Chemotherapy	151	22 (14.6%)	4 (2.6%)	32 (21.2%)	13 (8.6%)	25 (16.6%)	18 (11.9%)	22 (14.6%)	15 (9.9%)
Endocrine therapy	36	5 (13.8%)	1 (2.8%)	6 (16.7%)	4 (11.1%)	-	6 (16.7%)	1 (2.8%)	13 (36.1%)
Immuno-therapy	2	-	-	-	-	2 (100%)	-	-	-
Radiotherapy	68	10 (14.7%)	2 (2.9%)	13 (19.2%)	3 (4.4%)	8 (11.8%)	11 (16.2%)	9 (13.2%)	12 (17.6%)
Cortico-therapy	10	3 (30. 0%)	-	3 (30.0%)	1 (10.0%)	1 (10.0%)	1 (10. 0%)	-	1 (10.0%)
Total		33	7	38	18	35	33	56	47

**Table 4:** Average treatment duration (in months) by age group, medical center, and underlying disease.

Medical Center/Neoplasm	Overall Mean ± SD	Age Groups—Years Old (Mean ± SD)			
		22–54 Years Old	55–64 Years Old	65–71 Years Old	72–84 Years Old
Craiova	25.3 ± 17.7	15.8 ± 9.3	30.4 ± 20.9	23.6 ± 14.5	26.3 ± 18.9
Constanta	23.8 ± 14.4	18.4 ± 14.1	26.3 ± 13.7	25.2 ± 13.7	23.8 ± 15.9
Breast	24.7 ± 15.7	18.4 ± 12.5	30.5 ± 20.6	21.8 ± 9.6	28.4 ± 13.7
Prostate	26.1 ± 16.5	11.0 ± 0.0	31.1 ± 10.5	26.6 ± 14.9	24.2 ± 19.6
Others	22.2 ± 15.6	16.0 ± 14.2	20.4 ± 11.5	26.2 ± 17.5	22.1 ± 17.2
Mean ± SD	24.4 ± 15.8	17.6 ± 12.5	28.2 ± 17.2	24.6 ± 13.9	25.0 ± 17.2

**Table 5:** Demographic factors, the disease, and its therapy.

Category	Parameter	Value	N (%)/Mean ± SD		*p*
			Non-MRONJ	MRONJ	
Demographic factors	Gender	Female	51 (60.7%)	58 (64.4%)	0.611 *
		Male	33 (39.3%)	32 (35.6%)	
	Age (years old)	22–54	20 (23.8%)/47.1 ± 7.6	10 (11.1%)/46.4 ± 3.9	0.173 *
		55–64	20 (23.8%)/60.3 ± 3.0	26 (28.9%)/60.2 ± 2.5	
		65–71	23 (27.4%)/68.4 ± 2.1	27 (30.0%)/67.3 ± 2.1	
		72–84	21 (25.0%)/77.4 ± 4.1	27 (30.0%)/76.0 ± 3.2	
	Medical center	Craiova	20 (23.8%)	53 (58.9%)	<0.0005 *
		Constanta	64 (76.2%)	37 (41.1%)	
	Residency	Urban	64 (76.2%)	61 (67.8%)	0.218 *
		Rural	20 (23.8%)	29 (32.2%)	
	Smoking status	No	47 (56.0%)	53 (58.9%)	0.695 *
		Yes	37 (44.0%)	37 (41.1%)	
Cancer type	Primary diagnostic (neoplasm)	Breast	39 (46.4%)	43 (47.8%)	0.963 *
		Prostate	22 (26.2%)	24 (26.7%)	
		Other neoplasms	23 (27.4%)	23 (25.5%)	
		Pulmonary	11 (47.9%)	3 (13.0%)	-
		Myeloma	7 (30.6%)	2 (8.8%)	-
		Genital	0 (0.0%)	7 (30.5%)	-
		Digestive	1 (4.3%)	5 (21.7%)	-
		Renal	1 (4.3%)	4 (17.4%)	-
		Cerebral	1 (4.3%)	0 (0.0%)	-
		Bladder	1 (4.3%)	0 (0.0%)	-
		Spinal cord	0 (0.0%)	1 (4.3%)	-
		Pharynx	1 (4.3%)	0 (0.0%)	-
		Thyroid	0 (0.0%)	1 (4.3%)	-
Metastasis	Bone metastasis	No	19 (22.6%)	1 (1.1%)	<0.0005 *
		Yes	65 (77.4%)	89 (98.9%)	
Therapy	Chemotherapy	No	18 (21.4%)	5 (5.6%)	0.002 *
		Yes	66 (78.6%)	85 (94.4%)	
	Endocrine therapy	No	57 (67.9%)	81 (90.0%)	<0.0005 *
		Yes	27 (32.1%)	9 (10.0%)	
	Immunotherapy	No	83 (98.8%)	89 (98.9%)	0.734 **
		Yes	1 (1.2%)	1 (1.1%)	
	Radiotherapy	No	51 (60.7%)	55 (61.1%)	0.957 *
		Yes	33 (39.3%)	35 (38.9%)	
	Corticotherapy	No	74 (88.1%)	90 (100%)	0.001 **
		Yes	10 (11.9%)	0 (0%)	
BF	BF type	Zoledronic acid	84 (100%)	90 (100%)	-
	BF	Only Zoledronic acid	79 (94.0%)	90 (100%)	0.025 **
		Oral BF followed by Zoledronic acid	5 (6.0%)	0 (0.0%)	
Comorbidities	Cardiovascular diseases	No	55 (65.5%)	38 (42.2%)	0.420
		Yes	29 (34.5%)	52 (57.8%)	
	Hypertension	No	62 (73.8%)	48 (53.3%)	0.005 *
		Yes	22 (26.2%)	42 (46.7%)	
	Nutritional diseases	No	54 (64.3%)	64 (71.1%)	0.336
		Yes	30 (35.7%)	26 (28.9%)	
	Diabetes mellitus	No	77 (91.7%)	79 (87.8%)	0.400
		Yes	7 (8.3%)	11 (12.2%)	
	Obesity	No	72 (85.7%)	86 (95.6%)	0.025 *
		Yes	12 (14.3%)	4 (4.4%)	
	Anemia	No	67 (79.8%)	89 (98.9%)	<0.0005 *
		Yes	17 (20.2%)	1 (1.1%)	
	Renal diseases	No	67 (79.8%)	76 (84.4%)	0.420
		Yes	17 (20.2%)	14 (15.6%)	

* Chi-Square test. ** Fisher’s Exact test.

**Table 6:** Characteristics of MRONJ for patients included in the study group.

Parameter	Value	N (%)			*p*
		Overall	Craiova	Constanta	
MRONJ stage	2	71 (78.9%)	43 (82.0%)	28 (75.7%)	0.604 *
	3	19 (21.1%)	10 (18.0%)	9 (24.3%)	
Trigger factor	Periodontal disease	13 (14.4%)	10 (18.9%)	3 (8.1%)	0.351 *
	Periapical disease	26 (28.9%)	15 (28.3%)	11 (29.7%)	
	Extraction	51 (56.7%)	28 (52.8%)	23 (62.2%)	
Overall location	Upper jaw	28 (31.2%)	16 (30.2%)	12 (32.4%)	0.897 **
	Lower jaw	58 (64.4%)	35 (66.0%)	23 (62.2%)	
	Both jaws	4 (4.4%)	2 (3.8%)	2 (5.4%)	
Maxillary	Anterior	8 (25.0%)	3 (16.7%)	5 (35.7%)	-
	Posterior	24 (75.0%)	15 (83.3%)	9 (64.3%)	
Mandible	Anterior	13 (21.0%)	8 (21.6%)	5 (20.0%)	-
	Posterior	49 (79.0 %)	29 (78.4%)	20 (80.0%)	
Denudated bone	No	3 (3.3%)	1 (1.9%)	2 (5.4%)	0.360 **
	Yes	87 (96.7%)	52 (98.1%)	35 (94.6%)	
Hypoesthesia	No	74 (82.2%)	44 (83.0%)	30 (81.1%)	1.000 *
	Yes	16 (17.8%)	9 (17.0%)	7 (18.9%)	
Surgical treatment	No	6 (6.7%)	3 (5.7%)	3 (8.1%)	0.886 **
	Surgical intervention	76 (84.4%)	45 (84.9%)	31 (83.8%)	
	Relapse followed by another surgical intervention	8 (8.9%)	5 (9.4%)	3 (8.1%)	
Resection	No	69 (76.7%)	36 (67.9%)	33 (89.2%)	0.023 *
	Yes	21 (23.3%)	17 (32.1%)	4 (10.8%)	
Sequestrectomy and Curettage	No	19 (21.1%)	15 (28.3%)	4 (10.8%)	0.045 *
	Yes	71 (78.9%)	38 (71.7%)	33 (89.2%)	

* Chi-Square test. ** Fisher’s Exact test.

**Table 7:** Treatment duration (in months).

Parameter	Value (Months)	N (%)/Mean ± SD		Total	*p*
		Non-MRONJ	MRONJ		
Treatment duration	1–12	43 (82.7%)/8.0 ± 3.0	9 (17.3%)/9.2 ± 4.3	52 (100%)	<0.0005 *
	13–24	19 (31.7%)/17.7 ± 3.7	41 (68.3%)/22.5 ± 2.8	60 (100%)	
	25–36	9 (26.5%)/31.7 ± 3.7	25 (73.5%)/35.0 ± 2.3	34 (100%)	
	>36	13 (46.4%)/52.4 ± 19.9	15 (53.6%)/48.5 ± 6.3	28 (100%)	

* Chi-Square test.

**Table 8:** Binomial regression—results.

Parameter	*p*	Odds Ratio	95% CI for Odds Ratio *	
			Lower	Upper
Gender	0.515	1.380	0.524	3.637
Age	0.572	1.012	0.972	1.053
Smoking status	0.754	1.148	0.484	2.724
Duration of treatment	<0.005	1.701	1.319	2.194
Chemotherapy	0.007	7.529	1.744	32.507
Endocrine therapy	0.001	0.174	0.061	0.496
Radiotherapy	0.299	1.550	0.678	3.542
DM	0.660	1.350	0.354	5.145
Obesity	0.024	0.182	0.041	0.800
Hypertension	0.002	3.793	1.613	8.916

* 95% confidence interval. DM—diabetes mellitus.

## Data Availability

The authors declare that the data of this research are available from the corresponding authors upon reasonable request.

## Supplementary Materials

The following supporting information can be downloaded at: https://www.mdpi.com/article/10.3390/jcm12113747/s1, Table S1: Distribution of patients by medical center and age groups. Table S2: Distribution of patients by neoplasm, age groups and gender.

## Appendix A. Chemotherapy Medication

**Therapy**	**Medication**
CHEMOTHERAPY	DOCETAXEL
	PEMETREXED (ALIMTA)
	PACLITAXEL
	NAVELBIN
	CYCLOPHOSPHAMIDE
	CARBOPLATIN
	ETOPOSID
	MELPHALAN (ALKERAN)
	GEMCITABINE (GEMZAR)
	DOXORUBICIN
COMBINATION CHEMOTHERAPY	EC (EPIRUBICIN, CYCLOPHOSPHAMIDE)
	PACLITAXEL + NAVELBINE
	DOXORUBICIN + CYCLOPHOSPHAMIDE
ENDOCRINE THERAPY	GOSERELIN (ZOLADEX)
	ABIRATERON
	LETROZOLE
	RIBOCICLIB
	FULVESTRANT
	LEUPROLIDE ACETATE (ELIGARD)
	TAMOXIFEN
	BICALUTAMIDE
MOLECULAR-TARGETED THERAPY	PALBOCICLIB
	BORTEZOMIB
	ABEMACICLIB
	PERTUZUMAB
	TRASTUZUMAB(HERCEPTIN)
	OSIMERTINIB
	SUNITINIB (SUTENT)
	CABOZANTINIB
COMBINATION CHEMOTHERAPY/HORMONOTHERAPY AND TARGETED MOLECULAR THERAPY	ATEZOLIZUMAB + ETOPOSID + CRB
	CYBORD (CYCLOPHOSPHAMIDE + BORTEZOMIB + DEXAMETHASONE)
	TAMOXIFEN + ABEMACICLIB + FULVESTRANT
	PALBOCICLIB + FULVESTRANT
	PALBOCICLIB + LETROZOLE
AHT	ATEZOLIZUMAB
	PEMBROLIZUMAB
