# Supplementary material for: Risk Factors for Medication-Related Osteonecrosis of the Jaw—A Binomial Analysis of Data of Cancer Patients from Craiova and Constanta Treated with Zoledronic Acid
Source: J Clin Med. 2023 May 29;12(11):3747. doi: 10.3390/jcm12113747 (PMC10253558; doi:10.3390/jcm12113747)
Supplement: Supplementary file 1 [file jcm-12-03747-s001.zip › jcm-2352218-supplementary.pdf]

Distribution of patients by medical center, age groups, neoplasm and gender

Patients included in the study lot were distributed in age groups, expressed as following: 22-54 (young adults), 55-64 (mature adults), 65-71 (young old) and 72-84 years old (old old) (Table S1).

For the first two age groups, females were predominant, representing more than 70% of participants, while males were mostly included in the older age groups compared to females. Following a Mann-Whitney U test, the differences in gender distribution between age groups were statistically significant, $U = 4339$, $z = 2.662$, $p = 0.008$.

Almost three quarters of patients from the study group (125 patients, representing 71.8%) lived in urban areas (with a male/female ratio of approximately 1/3), while 49 patients (28.2%) lived in rural areas (with a similar gender distribution).

All 174 patients were previously diagnosed with neoplasms, of which breast and prostate cancers were predominant in our study group. Overall, 82 females had breast cancer (47.2%), 46 males had prostate cancer (26.4%), while the rest of 46 patients had other neoplasms: pulmonary, myeloma, genital, digestive, renal, cerebral, bladder, spinal cord, pharynx, thyroid. Bone metastases were diagnosed in 154 patients (88.5%, almost 2/3 being females, mostly encountered for ages above 65 years old) (Table S2).

Table S1. Distribution of patients by medical center and age groups.

**Medical center**	**Total N (%)**	**Age groups N (%) – years old (mean ± SD)**			
		**22-54**	**55-64**	**65-71**	**72-84**
**Craiova**	73 (100)	10 (13.7%)<br>45.2 ± 3.6	21 (28.8%)<br>60.4 ± 2.6	20 (27.4%)<br>67.2 ± 1.8	22 (30.1%)<br>76.6 ± 3.8
**Constanta**	101 (100)	20 (19.8%)<br>47.7 ± 7.5	25 (24.8%)<br>60.2 ± 2.8	30 (29.7%)<br>68.2 ± 2.2	26 (25.7%)<br>76.6 ± 3.7
**Total**	**174**	**30**	**46**	**50**	**48**

Table S2. Distribution of patients by neoplasm, age groups and gender.

**Neoplasm**	**Total N**	**Age groups / gender - Number of patients (%)**							
		**22-54**		**55-64**		**65-71**		**72-84**	
		F	M	F	M	F	M	F	M
Breast	82	22 (26.8%)	-	25 (30.5%)	-	20 (24.4%)	-	15 (18.3%)	-
Prostate	46	-	1 (2.2%)	-	10 (21.7%)	-	14 (30.4%)	-	21 (45.7%)
Others	46	3 (6.5%)	4 (8.7%)	8 (17.4%)	3 (6.5%)	7 (15.2%)	9 (19.6%)	9 (19.6%)	3 (6.5%)
Pulmonary	14	1 (7.1%)	2 (14.3%)	-	2 (14.3%)	3 (21.4%)	4 (28.6%)	2 (14.3%)	-
Myeloma	9	-	1 (11.1%)	2 (22.2%)	-	1 (11.1%)	2 (22.2%)	3 (33. 3%)	-
Genital	7	-	-	4 (57.1%)	-	1 (14.3%)	-	2 (28.6%)	-
Digestive	6	-	-	2 (33.3%)	-	1 (16.7%)	1 (16.7%)	1 (16.7%)	1 (16.7%)
Renal	5	-	1 (20.0%)	-	1 (20.0%)	1 (20.0%)	1 (20.0%)	-	1 (20.0%)
Bladder	1	-	-	-	-	-	-	-	1 (100%)
Cerebral	1	-	-	-	-	-	1 (100%)	-	-
Spinal cord	1	-	-	-	-	-	-	1 (100%)	-
Pharynx	1	1 (100%)	-	-	-	-	-	-	-

Thyroid	1	1 (100%)	-	-	-	-	-	-	-
Bone metastasis	154	21 (13.6%)	4 (2.6%)	27 (17.5%)	13 (8.4%)	23 (14.9%)	20 (13.0%)	23 (14.9%)	23 (14.9%)
**Total**		**49**	**13**	**68**	**29**	**57**	**52**	**56**	**50**
